# In Vitro Metabolic Transformation of Pharmaceuticals by Hepatic S9 Fractions from Common Carp *(Cyprinus carpio)*

**DOI:** 10.3390/molecules25112690

**Published:** 2020-06-10

**Authors:** Viktoriia Burkina, Sidika Sakalli, Pham Thai Giang, Kateřina Grabicová, Andrea Vojs Staňová, Galia Zamaratskaia, Vladimir Zlabek

**Affiliations:** 1 South Bohemian Research Center of Aquaculture and Biodiversity of Hydrocenoses, Faculty of Fisheries and Protection of Waters, University of South Bohemia in Ceske Budejovice, Zatisi 728/II, 389 25 Vodňany, Czech Republic; sakalli@frov.jcu.cz (S.S.); ptgiang@ria1.org (P.T.G.); grabicova@frov.jcu.cz (K.G.); vojsstanova@frov.jcu.cz (A.V.S.); galia.zamaratskaia@slu.se (G.Z.); vzlabek@frov.jcu.cz (V.Z.); 2 Department of Molecular Sciences, Swedish University of Agricultural Sciences, P.O. Box 7015, SE-750 07 Uppsala, Sweden; 3 Research Institute for Aquaculture No 1, Dinh Bang 220000, Tu Son, Bac Ninh, Vietnam; 4 Department of Analytical Chemistry, Faculty of Natural Sciences, Comenius University in Bratislava, Ilkovicova 6, SK-842 15 Bratislava, Slovakia

**Keywords:** cytochrome P450, metabolite formation, citalopram, sertraline, venlafaxine, metoprolol, environmental toxicology

## Abstract

Water from wastewater treatment plants contains concentrations of pharmaceutically active compounds as high as micrograms per liter, which can adversely affect fish health and behavior, and contaminate the food chain. Here, we tested the ability of the common carp hepatic S9 fraction to produce the main metabolites from citalopram, metoprolol, sertraline, and venlafaxine. Metabolism in fish S9 fractions was compared to that in sheep. The metabolism of citalopram was further studied in fish. Our results suggest a large difference in the rate of metabolites formation between fish and sheep. Fish hepatic S9 fractions do not show an ability to form metabolites from venlafaxine, which was also the case for sheep. Citalopram, metoprolol, and sertraline were metabolized by both fish and sheep S9. Citalopram showed concentration-dependent *N*-desmethylcitalopram formation with V_max_ = 1781 pmol/min/mg and K_m_ = 29.7 μM. The presence of ellipticine, a specific CYP1A inhibitor, in the incubations reduced the formation of *N*-desmethylcitalopram by 30–100% depending on the applied concentration. These findings suggest that CYP1A is the major enzyme contributing to the formation of *N*-desmethylcitalopram. In summary, the results from the present in vitro study suggest that common carp can form the major metabolites of citalopram, metoprolol, and sertraline.

## 1. Introduction

The contamination of aquatic systems by human pharmaceuticals and personal care products is increasingly concerning. The use of pharmaceuticals is often essential in supporting public health, treating and curing diseases, and easing symptoms. However, once pharmaceuticals have been absorbed by the host organism and excreted as the parent compound, their metabolites, or both, can be transferred into wastewater treatment plant (WWTP) facilities. Currently, such facilities do not have additional steps to remove pharmaceutically active compounds. Therefore, WWTPs are recognized as hot spots for releasing pharmaceuticals into aquatic environments [1,2]. These compounds are considered as pseudo-persistent compounds in WWTP effluents due to regular prescription and continuous use. This contributes to chronic exposure of aquatic organisms, such as fish, to human pharmaceuticals. Even though surface water concentrations typically contain pharmaceutical compounds in the nanogram to microgram per litter range [3,4], chronic exposure will likely cause adverse effects on non-target organisms [5,6,7]. The accumulation of pharmaceutical compounds in the environment can indirectly affect public health through the food chain [8,9]. The south region of the Czech Republic continues to farm common carps by traditional methods in ponds. Some of these ponds are connected through a cascade system with a WWTP outlet. Thus, the WWTP of Vodňany town (~7000 inhabitants) discharges treated water directly into the pond, where common juvenile carps are restocked. Environmental risk assessments for this pond system have shown that the presence of pharmaceuticals and their metabolites affected hepatic and intestinal CYP enzymes, fatty acid metabolism, and intestinal microbiome in common carp [10,11].

WWTPs can act as a channel by which many human 44 pharmaceuticals are further released into the environment through effluent discharges [12]. Human pharmaceuticals and their metabolites have been detected in WWTP influent and effluent [13,14]. During the treatment processes at the WWTP, some of pharmaceuticals are completely removed, and some only partly removed, with the formation of transformation product. These transformation products might present a risk for ecosystem contamination [15].

Fish is a good indicator of the presence of pharmaceuticals in water, based on their low capacity to metabolize and then eliminate them [16]. However, under well-controlled experimental conditions, when fish are kept in water tanks, and pure pharmaceuticals (e.g., diltiazem) are applied to water for research purposes, the presence of metabolites after a certain time was also detected in water [17,18]. Therefore, in in situ tests in cascade ponds in Vodňany town, citalopram, metoprolol, sertraline, venlafaxine, and their metabolites have been detected in common carp fish hepatic tissue, water, and soil [19,20]. It is uncertain whether the metabolites, which have been previously detected in the pond, are at least partly produced during hepatic metabolism in fish and excreted back to the water. The discharge scenario of pharmaceuticals release from the WWTP of Vodňany town into the pond system is typical of many European countries, and thus, understanding the ability of fish to metabolize these compounds is highly relevant [21].

The pharmaceuticals exert their effects by binding to specific proteins and receptors [22]. Similar to mammalians, the piscine cytochrome P450 (CYP) system is the major enzyme in xenobiotic metabolism [23]. Metabolic pathways of some xenobiotics were characterized using piscine in vitro models [24,25]. In vitro metabolism of pharmaceutical compounds is commonly studied in liver microsomes because they contain a high amount of CYPs, flavin-containing monooxygenases, and uridine glucuronide transferase. The use of S9 fractions, which contain both phases I and II enzymes, allows investigation of the entire metabolic fate of compounds in conditions that more closely resemble the physiological condition than when using microsomes [26].

Amino acid sequence of ovine CYP1A1 shows high identity homology of CYP1A1 with other often used mammalian models like rat (82%, [27]), and pig (>80%, [28]) and even with marine mammals including minke whale (88%, [28]).

Here we aimed to characterize the formation of the main metabolites of four commonly occurred pharmaceuticals, and determine if the CYP1A1 isoform is involved in their metabolism using common carp liver S9 fractions. The study was focused on pharmaceuticals and their main metabolites previously found in fish from Cezarka pond, which are affected by treated wastewater.

## 2. Results

Given that, in this study, fish were taken from pond, which receives water from the river, fish hepatic tissue were characterized with respect to the presence of 72 pharmaceuticals and 6 metabolites, whose presence is suspected in pond water. The concentrations of all pharmaceuticals in liver tissue were negligible and confounding factor can be excluded (Supplementary material 1).

### 2.1. Characterization of Piscine and Ovine S9 Fraction

Common carp S9 fraction was initially characterized on activity of phase I enzymes, by using of known substrates, for CYP1A1 and CYP3A-like activity (Table 1). As an additional confirmation of S9 activity, formation of the tris(*n*-butyl) phosphate (TNBP) metabolite was observed (data not showed). The metabolite formation was not observed in the incubations without S9 fraction or without substrate.

### 2.2. Metabolites Formation

The formation of the main metabolite of four pharmaceuticals was investigated in the hepatic S9 fraction of sheep, common carps, and carps injected with 50 mg/kg with BNF to induce CYP1A1 activity. The metabolite formation was observed in optimized conditions.

The formation of metabolites was observed in incubations with citalopram, metoprolol, and sertraline after 60 min incubation with piscine not induced and induced S9 fractions (Table 2). In addition to those three compounds, the sheep S9 fraction produced the *O*-desmethylvenlafaxine metabolite. The *N*-desmethylcitalopram and norsertraline metabolite formation were 5.3- and 1.7-fold higher in induced fish than in the not induced S9 fraction, while the formation of metoprolol acid was reduced by 3.4-fold. Sheep metabolic activity was evidently higher than in fish induced with BNF.

### 2.3. N-desmethylcitalopram Formation

The kinetics of metabolite formation was assessed only for the citalopram compound, since the formation of metoprolol acid and norsertraline was not non-linear within applied substrate concentrations.

**Figure 1:**
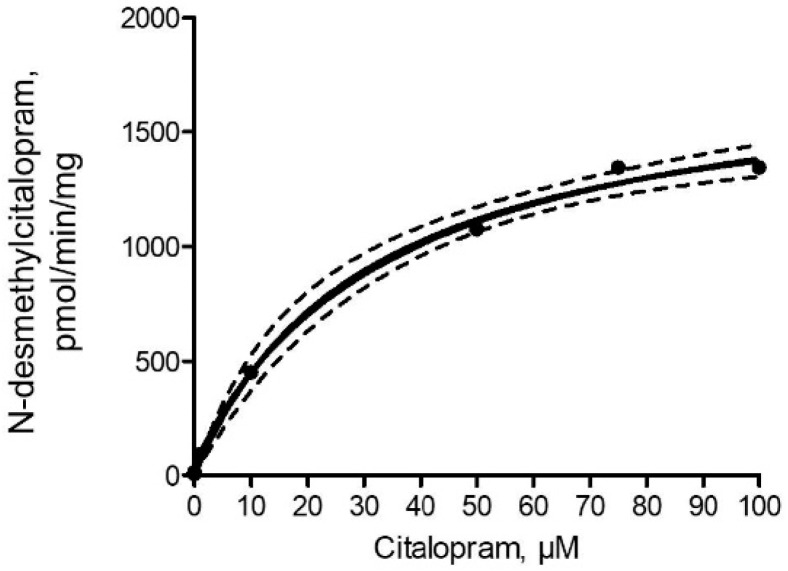
Saturation curve for *N*-desmethylcitalopram formation from citalopram in S9 fractions from the liver of common carp (*n* = 12 in 3 pools with equal sex ratio). The dashed line indicates a 95% confidence level of the best-fit curve.

The kinetics of *N*-desmethylcitalopram was best described by the Michaelis–Menten equation (r^2^ = 0.997; Figure 1). The maximum reaction speed (V_max_) and Michaelis constant (K_m_) of *N*-desmethylcitalopram formation estimated from fitting to Michaelis-Menten equation were 1781 ± 90 pmol/min/mg and 29.7 ± 4.6 μM, respectively.

### 2.4. Inhibition Study

The rates of *N*-desmethylcitalopram formation from citalopram (5 μM) in the presence of inhibitor ellipticine (data not shown) show that 0.125 μM ellipticine reduced *N*-desmethylcitalopram formation by 30 ± 10%, while 1.25 and 12.5 μM completely inhibited *N*-desmethylcitalopram formation in the S9 fraction from common carp.

## 3. Discussion

Selected pharmaceuticals are widely prescribed in European countries [29,30] and have been routinely reported in different sample matrices, like sediment, sludge, soil, plants, and fish. Eighteen pharmaceuticals and seven metabolites have been identified in water and sediments from the pond, which receives discharged water from the WWTP of Vodňany town [20]. Fish living in that pond show the presence of 14 pharmaceuticals, including four parent compounds and their main metabolites (*N*-desmethylcitalopram, norsertraline, metoprolol acid, and *O*-desmethylvenlafaxine) in hepatic tissues [19,20]. However, it is not known whether these metabolites were taken up from the surrounding aquatic environment or produced by fish from the original compounds. To the best of our knowledge, this is the first study to report that hepatic S9 fractions of common carp were able to produce *N*-desmethylcitalopram, norsertraline, and metoprolol acid metabolites from citalopram, sertraline, and metoprolol, respectively. The use of a specific inhibitor of CYP1A suggested that CYP1A is involved in phase I biotransformation of citalopram in fish.

In the present study, metoprolol, sertraline, and citalopram showed metabolite formation by the common carp hepatic S9 fraction. It is well-documented that these pharmaceuticals are metabolized in mammals by CYPs. For example, formation of desmethylcitalopram is catalyzed by the isoenzymes CYP2C19, CYP3A4, and CYP2D6 [31,32], formation of metoprolol acid by CYP2D6 [33] and norsertraline formation with CYP2C9, CYP3A4, CYP2C19, CYP2D6, and CYP2B6 [34,35,36] in mammals. To date, results from the studies on pharmaceutical compounds metabolism in fish (both in vivo and in vitro) remain controversial. It was suggested that after chronic exposure of diltiazem to rainbow trout, diltiazem metabolites had been identified in hepatic tissues of rainbow trout [18], contrary to Connors et al. [37], who could not find any diltiazem substrate depletion by the rainbow trout liver S9 fraction.

The contribution of piscine CYPs to biotransformation of pharmaceuticals is less described compared to in mammalians. It is likely that some differences exist. Thus, venlafaxine is attributed by CYP2D6, CYP2C19, and CYP3A4 in humans [38]. Fish S9 fractions are lacking activity toward the prototypical human CYP2C substrates [23]. Earlier, it was shown that venlafaxine did not alter CYP1A and CYP3A-like mediated reactions, such as ethoxyresorufin-O-deethylase (EROD) and benzyloxy-4-trifluoromethylcoumarin-O-debenzyloxylase (BFCOD) activity in the rainbow trout microsomal fraction [39]. Based on these results, we could not find the main venlafaxine metabolite by carp S9. Therefore, venlafaxine and its metabolite, which were found in fish tissue from the pond system, were removed from the water. Venlafaxine and *O*-desmethylvenlafaxine could be bioavailable in the fish circulation system and provoke adverse effects, which is connected with changes in behavioral performance [40].

Citalopram is a selective serotonin reuptake inhibitor medication and is one of the most prescribed and used antidepressants in the world [41], due to its low potential to show clinically relevant drug-drug interactions, lower binding affinity to serum proteins, and low cardiac toxicity compared to other antidepressants [42,43,44]. Citalopram and its metabolite *N*-desmethylcitalopram were found in different environmental matrices (water, sediment, and fish) of the pond which received discharged water from WWTP of Vodňany town [20]. The average concentration of citalopram and *N*-desmethylcitalopram found in all analyzed hepatic tissue of common carp was 3.1 ± 2.9, and 2.6 ± 1.4 ng/g wet weight, respectively [19]. The major metabolic pathway of citalopram was previously described by von Moltke et al. [45], where biphasic plots for citalopram *N*-demethylation with V_max_ 25 and 158 pmol/min/mg and K_m_ 17.3 and 184.9 μM for at least two enzymes, respectively, was shown using human hepatic microsomes. However, when data were placed on the monophasic model, V_max_ reached 183–281 pmol/min/mg for *N*-desmethylcitalopram. Further formation of *N*-desmethylcitalopram indicated that CYP3A4, 2C19, and 2D6 contribute *N*-demethylation of citalopram [45]. In the present study, Michaelis-Menten parameters of formation of *N*-desmethylcitalopram by the fish hepatic S9 fraction were 1781 pmol/min/mg K_m_ 29.7 μM. The further involvement of CYP1A1 enzymes in the metabolism of citalopram was investigated using the selective chemical inhibitor ellipticine. Our inhibition study showed that ellipticine decreased the formation of *N*-desmethylcitalopram, suggesting that CYP1A protein is involved in citalopram metabolism in fish.

Fish CYP1A is the most studied xenobiotic-metabolizing enzyme in phase I due to its toxicological importance. This enzyme can be induced or inhibited by several xenobiotics [46], altering the toxicity of chemical contaminants. BNF is a well-known agonist of the aryl hydrocarbon receptor, which results in an increase of transcription of CYP1A and CYP1A enzyme activity. Since the exposure of fish to environmental contaminants can result in the induction of CYP1A, thus, different outcomes might arise for pharmaceuticals metabolized typically or mainly by this isoform. In the present study, we tested whether fish, induced with BNF, can show different patterns of metabolite formation due to the interaction of parent pharmaceutical compounds and elevated CYP1A1. The *N*-desmethylcitalopram, norsertraline, and metoprolol acid metabolite formation differed in the S9 fraction from common carp induced with BNF. A concentration greater than 5-times concentration that of *N*-desmethylcitalopram was formed by S9 fraction from BNF induced fish, suggesting synergistic or additive effect.

This study contributes to the current knowledge on pharmaceutical bioaccumulation and pharmacokinetics. In summary, fish can generate the main metabolites of metoprolol, sertraline, and citalopram during hepatic metabolism. The inhibition data indicated that the co-administration of citalopram with other pharmaceuticals from water might suggest inhibition of at least the piscine CYP1A1. To evaluate the potential adverse effects in aquatic organisms, further studies are now needed that combine pharmaceuticals with different pharmacological effects. Furthermore, role of phase II metabolic reactions should be explored.

## 4. Materials and Methods

### 4.1. Chemicals and Reagents

Chemicals were purchased from multiple companies at the highest available purity. Citalopram, sertraline, and venlafaxine were purchased from AK Scientific (Union City, NJ, USA); metoprolol and tris(*n*-butyl) phosphate (TNBP) from Sigma Aldrich (Darmstadt, Germany); metabolites norsertraline and *N*-desmethylcitalopram from Labicom (Olomouc, The Czech Republic); metoprolol acid from Toronto Research Chemicals and *O*-desmethylvenlafaxine; and chemicals for preparation of S9 fractions and performing biochemical reactions from Sigma Aldrich (Darmstadt, Germany). An analytical procedure was performed using internal standards (IS). An isotope-labelled carbamazepine (D10) was obtained from CDN Isotopes (Pointe-Claire, QC, Canada), citalopram (D6), venlafaxine (D6) and sertraline (D3) were purchased from Lipomed AG (Arlesheim, Switzerland), and metoprolol (D7) was obtained from Alsachim (Strasbourg, France). Chemical reaction compartments nicotinamide-adenine dinucleotide phosphate (NADPH), uridine 5′- diphosphoglucuronic acid (UDPGA), resorufin, 7-ethoxyresorufin, 7-benzyloxy-4-trifluoromethylcoumarin (BFC), 7-hydroxy-4-trifluoromethylcoumarin (HFC) and ellipticine were purchased from Sigma Aldrich (Darmstadt, Germany). Organic solvents for HPLC-MS (LC-MS grade) were purchased from Merck (Darmstadt, Germany). Citalopram, metoprolol, sertraline, and venlafaxine stocks for the experiment were prepared at concentrations of 20 mM in methanol and stored at −20 °C until use.

### 4.2. Animals

Fish. Fish were collected from a fishpond with a low concentration level of pharmaceuticals (305–880 ng/POCIS) [10]. Thus, fish were stocked in the pond for 1 year with a natural photoperiod. The fish were allowed to eat and swim freely in the pond without interaction with humans and avoiding stress conditions. In total, 1-year-old common carps (*n* = 12) weighing 695 ± 116 g (mean ± standard deviation) and length 330 ± 19 mm were collected during spring 2018. Additionally, β-naphtaflavone (BNF) was administrated into four fish at levels of 50 mg/kg of BNF in corn oil. Following administration, individuals were kept in a separate aquaria for 48 h, in order to achieve CYP1A1 induction. The fish were then sacrificed according to the ethical rules of the EU harmonized Animal Welfare Act of the Czech Republic. The unit is licensed (No. 53100/2013-MZE-17214), according to the Czech National Directive (the Law against Animal Cruelty, No. 246/1992). Before sampling, fish were anesthetized in an ice bath, and their spinal cords were immediately cut. Fish was bloodless and hepatic tissues were collected and stored at −80 °C before use for the preparation of S9 fraction.

Sheep. Hepatic tissue from one male and one female Suffolk sheep (age, 2 years; weight 70–80 kg) were collected at a slaughterhouse in Vodňany town, Czech Republic, and stored at −80 °C. Sheep is a relatively new animal model, which has started to be used in veterinarian investigations related to drug-drug interactions, as well as for effective therapy and the limitation of drug-resistance development. Sheep phase I biotransformation enzymes, including CYPs-dependent metabolic activities in sheep liver, were already characterized by Szotakova et al. [47], Maté et al. [48] and Stuchlíková et al. [49]. The use of the S9 fraction from sheep hepatic tissue could represent the mammalian metabolic system in relation to selected pharmaceuticals.

### 4.3. Preparation of S9 Fraction

Hepatic tissue (approx. 1.2 g) of twelve individual common carps and two individual sheep were homogenized in 10 mM Tris–HCl buffer, pH 7.4, containing 250 mM sucrose separately using an T-25 Ultra Turrax homogenizer Ika (Staufen, Germany) followed by centrifugation at 10,000× *g* for 15 min at 4 °C to obtain S9 fractions. The protein concentration in S9 fractions was determined by the colorimetric method according to Smith et al. [50] at 595 nm. The S9 fractions were diluted to 10 mg/mL protein content. To account for the gender effect, four piscine S9 fractions were combined in one pool with the same ratio of males/females (1:1). The incubations were performed using three S9 pools from common carp. One pool was prepared from ovine S9 fractions.

### 4.4. S9 Fraction Characterization

The EROD and BFCOD activity were measured using black 96-well plate [51]. Individual standard curves of resorufin and HFC were used to calculate amount of product, which was produced in one minute by one mg of protein. The EROD incubation mixtures consisting of 0.2 mg of protein, 2 µM of 7-ethoxyresorufin, incubation medium (50 mM potassium phosphate buffer, pH 7.4), and 1 mM NADPH. The BFCOD incubation mixtures consisting of 0.2 mg of protein, 150 µM of BFC, incubation medium (50 mM potassium phosphate buffer, pH 7.4), and 0.5 mM NADPH. The total reaction volume was 260 mL in each well.

### 4.5. *In Vitro* Incubation of S9 Fraction with Pharmaceutical Compounds

In this in vitro study, applied concentration of pharmaceutical compounds was 1000 times higher than the concentration detected in common carp liver (0.4–3 ng/g) and blood plasma (0.13–0.24 ng/g) from in situ experiment in Cezarka pond [19].

To investigate the formation of metabolites, one concentration of pharmaceutical (2 μM) was incubated with 1 mg of protein in piscine or ovine S9 fraction, 1 mM NADPH, and 0.3 mM UDPGA in a potassium phosphate buffer (50 mM, 7.4 pH) (Table 2). The total volume of each incubation was 0.5 mL. The incubation mixture was vortexed and incubated for 1 h in a water bath (at 21 °C for fish and 37 °C for ovine). Then, the reaction was stopped by the addition of 0.5 mL ice-cold methanol, vortexed, and centrifuged at 10,000× *g* for 10 min. Four types of control incubation with no substrate or with no S9 protein fractions were also prepared to confirm the absence of interfering compounds and to identify any non-metabolically formed compounds. Sheep hepatic S9 fraction was used as a positive control, because sheep S9 fractions are known to metabolise the studied compounds. Additionally, control incubations (Table 3), with active and heat inactivated piscine and ovine S9 fractions, were conducted. The supernatant was filtrated (0.45 μm regenerated cellulose filter) and the internal standard for chemical analyses was added. According to Hou et al. [52], *Carassius carassius* hepatic microsomes can metabolize TNBP in vitro. Therefore, in this study, TNBP was selected as a compound to confirm the ability of common carp S9 fractions to form metabolites. S9 fractions with TNBP were incubated under the same conditions as other pharmaceuticals. The choice of optimal conditions for incubations with regards to the linearity of incubation time and protein concentrations was based on a previous study at our laboratory [24]. The kinetics of metabolite formation was continued only with a compound that shows the linear range for the rate of metabolite formation by fish S9 fractions.

### 4.6. The Kinetic Study Focused on Citalopram

Kinetic analysis using multiple substrate concentrations was conducted only with citalopram using the same procedure as above-described. The substrate concentrations were 0.01, 0.03, 0.1, 1, 10, 50, 75, and 100 μM of citalopram.

### 4.7. Inhibition Study Focused on Citalopram

Ellipticine (a specific inhibitor of mammalian [53] and piscine [54] CYP1A1) was used to investigate if CYP1A1 was responsible for metabolite formation. Incubations were performed only with citalopram. Three concentrations of ellipticine were used in the final incubations: 0.125, 1.25, and 12.5 μM. The choice of these concentrations was based on the previous results [25]. The degree of inhibition was assessed by comparison of metabolite formation in the presence and absence of inhibitor. The incubation without inhibitor contained the same amount of methanol as in the incubations with inhibitor (0.5% from total incubation volume).

### 4.8. Liquid Chromatography-High Resolution Mass Spectrometry (LC-HRMS)

The concentrations of the parent compound and its main metabolites (metoprolol acid, *N*-desmethylcitalopram, norsertraline, and *O*-desmethylvenlafaxine) were determined by LC HRMS (Thermo Fisher Scientific, San Jose, CA, USA). The chromatographic separation was performed on a Hypersil Gold aQ analytical column (50 × 2.1 mm; 5 μm particles, Thermo Fisher Scientific). The mobile phase consisted of solvent A (water acidified with 0.1% formic acid) and solvent B (acetonitrile with 0.1% formic acid). The gradient was set as follows: 0–1 min 100% A, 1–4 min decrease to 75% A, with 350 μL/min flow rate; then 4–8 min 40% A, 8–10 min 0% A, 10–12 min 0% A with 450 μL/min flow rate; then 12.05–15 min 100% A with 350 μL/min flow rate. A heated electrospray ionization (HESI) source was used for the ionization of the target compounds with a spray voltage of 3.5 kV and nitrogen as the sheath gas (40 arbitrary units), auxiliary gas (10 arbitrary units), and collision gas. The full scan mode with high resolution (70000 FWHM) in range 100–750 *m*/*z* was used with the AGC target 3e6, and the maximum filling time 50 ms. Data acquisition was performed with Xcalibur 4.3 Software, and data processing was performed using TraceFinder 3.3 Software (both Thermo Fisher Scientific, San Jose, CA, USA). Internal standard and matrix matching standard methods were used for the quantification of target analytes.

### 4.9. Data Analysis

The data were analyzed using non-linear regression analysis (GraphPad Prism version 4.0 for Windows, GraphPad Software, San Diego, CA, USA). The data were fitted to the Michaelis-Menten equation and two enzyme models. The goodness of fit was assessed by comparison of the coefficient of determination (r^2^). Visual analysis of Eadie-Hofstee plots was used to estimate whether one or more enzymes participate in the reaction.

## Figures and Tables

**Table 1:** Common carp hepatic S9 characterization biomarkers. Data are present as least squares mean ± SE, *n* = 12.

Biomarker	Units	Fish
EROD	pmol/min/mg	5.33 ± 1.99
BFCOD	pmol/min/mg	5.55 ± 3.58
TNBP		+

EROD—ethoxyresorufin O-dealkylation; BFCOD—7-benzyloxy-4-(trifluoromethyl) coumarin O-debenzyloxylase; TNBP—tris(*n*-butyl) phosphate; “+”—indicator of tri-iso-butyl phosphate metabolite production from TNBP.

**Table 2:** Concentrations of metabolites formed under the tested conditions.

	Metabolites Concentration, pmol/min/mg				LOQ ^1^ Min-Max, pmol/min
	Control (without S9)	Sheep S9	Fish S9	BNF ^1^ Fish S9	
*N*-desmethylcitalopram	<LOQ	2309	295	1557	8–23
Metoprolol acid	<LOQ	443	274	81	11–27
Norsertraline	<LOQ	3251	405	684	68–217
*O*-desmethylvenlafaxine	<LOQ	823	<LOQ	<LOQ	10–24

^1^ LOQ — limit of quantification; BNF — β-naphtaflavone.

**Table 3:** Incubation procedure of piscine or ovine S9 fractions to study formation of major metabolites of selected pharmaceuticals.

Incubation Type	Buffer	Pharmaceutical	S9 fraction	Co-Factors	Pharmaceutical Concentration
Control	+	-	+ piscine or ovine	+	-
Control	+	-	+ piscine or ovine	+	-
Control	+	-	+ piscine or ovine	+	-
Control	+	+	-	+	2 µM of citalopram or metoprolol or TNBP or sertraline or venlafaxine
Test	+	+	+ piscine or ovine	+	

“+”—indicator of added compound to incubation; “-“—indicator of absence compound in incubation; TNBP—tris(*n*-butyl) phosphate.

## Supplementary Materials

The supplementary material 1 is available online at https://zenodo.org/deposit/3833659.

## References

[1] Stroski K.M., Luong K.H., Challis J.K., Chaves-Barquero L.G., Hanson M.L., Wong C.S. (2020). Wastewater sources of per- and polyfluorinated alkyl substances (PFAS) and pharmaceuticals in four Canadian Arctic communities. Sci. Total Environ..

[2] Tröger R., Köhler S.J., Franke V., Bergstedt O., Wiberg K. (2020). A case study of organic micropollutants in a major Swedish water source – Removal efficiency in seven drinking water treatment plants and influence of operational age of granulated active carbon filters. Sci. Total Environ..

[3] Batt A.L., Kincaid T.M., Kostich M.S., Lazorchak J.M., Olsen A.R. (2016). Evaluating the extent of pharmaceuticals in surface waters of the United States using a National-scale Rivers and Streams Assessment survey. Environ. Toxicol. Chem..

[4] Petrie B., Barden R., Kasprzyk-Hordern B. (2015). A review on emerging contaminants in wastewaters and the environment: Current knowledge, understudied areas and recommendations for future monitoring. Water Res..

[5] Du S.N.N., Choi J.A., McCallum E.S., McLean A.R., Borowiec B.G., Balshine S., Scott G.R. (2019). Metabolic implications of exposure to wastewater effluent in bluegill sunfish. Comp. Biochem. Physiol. C-Toxicol. Pharmacol..

[6] McCallum E.S., Nikel K.E., Mehdi H., Du S.N.N., Bowman J.E., Midwood J.D., Kidd K.A., Scott G.R., Balshine S. (2019). Municipal wastewater effluent affects fish communities: A multi-year study involving two wastewater treatment plants. Environ. Pollut..

[7] Simmons D.B.D., McCallum E.S., Balshine S., Chandramouli B., Cosgrove J., Sherry J.P. (2017). Reduced anxiety is associated with the accumulation of six serotonin reuptake inhibitors in wastewater treatment effluent exposed goldfish *Carassius Auratus*. Sci. Rep..

[8] Brooks B.W., Chambliss C.K., Stanley J.K., Ramirez A., Banks K.E., Johnson R.D., Lewis R.J. (2005). Determination of select antidepressants in fish from an effluent-dominated stream. Environ. Toxicol. Chem..

[9] Huerta B., Rodriguez-Mozaz S., Lazorchak J., Barcelo D., Batt A., Wathen J., Stahl L. (2018). Presence of pharmaceuticals in fish collected from urban rivers in the U.S. EPA 2008–2009 National Rivers and Streams Assessment. Sci. Total Environ..

[10] Giang P.T., Burkina V., Sakalli S., Schmidt-Posthaus H., Rasmussen M.K., Randak T., Grabic R., Grabicova K., Fedorova G., Koba O. (2019). Effects of multi-component mixtures from sewage treatment plant effluent on common carp (*Cyprinus carpio*) under fully realistic condition. J. Environ. Manag..

[11] Sakalli S., Giang P.T., Burkina V., Zamaratskaia G., Rasmussen M.K., Bakal T., Tilami S.K., Sampels S., Kolarova J., Grabic R. (2018). The effects of sewage treatment plant effluents on hepatic and intestinal biomarkers in common carp (*Cyprinus carpio*). Sci. Total Environ..

[12] Luo Y., Guo W., Ngo H.H., Nghiem L.D., Hai F.I., Zhang J., Liang S., Wang X.C. (2014). A review on the occurrence of micropollutants in the aquatic environment and their fate and removal during wastewater treatment. Sci. Total Environ..

[13] Couto C.F., Lange L.C., Amaral M.C.S. (2019). Occurrence, fate and removal of pharmaceutically active compounds (PhACs) in water and wastewater treatment plants—A review. J. Water Process. Eng..

[14] Golovko O., Kumar V., Fedorova G., Randak T., Grabic R. (2014). Seasonal changes in antibiotics, antidepressants/psychiatric drugs, antihistamines and lipid regulators in a wastewater treatment plant. Chemosphere.

[15] Archer E., Petrie B., Kasprzyk-Hordern B., Wolfaardt G.M. (2017). The fate of pharmaceuticals and personal care products (PPCPs), endocrine disrupting contaminants (EDCs), metabolites and illicit drugs in a WWTW and environmental waters. Chemosphere.

[16] Wassenaar P.N.H., Verbruggen E.M.J., Cieraad E., Peijnenburg W.J.G.M., Vijver M.G. (2020). Variability in fish bioconcentration factors: Influences of study design and consequences for regulation. Chemosphere.

[17] Koba O., Steinbach C., Kroupova H.K., Grabicova K., Randak T., Grabic R. (2016). Investigation of diltiazem metabolism in fish using a hybrid quadrupole/orbital trap mass spectrometer. RCM.

[18] Steinbach C., Grabic R., Fedorova G., Koba O., Golovko O., Grabicova K., Kroupova H.K. (2016). Bioconcentration, metabolism and half-life time of the human therapeutic drug diltiazem in rainbow trout. Chemosphere.

[19] Grabicova K., Vojs Staňová A., Koba Ucun O., Borik A., Randak T., Grabic R. (2018). Development of a robust extraction procedure for the HPLC-ESI-HRPS determination of multi-residual pharmaceuticals in biota samples. Analytica Chimica Acta.

[20] Koba O., Grabicova K., Cerveny D., Turek J., Kolarova J., Randak T., Zlabek V., Grabic R. (2018). Transport of pharmaceuticals and their metabolites between water and sediments as a further potential exposure for aquatic organisms. J. Hazard. Mater..

[21] Fernández-Rubio J., Rodríguez-Gil J.L., Postigo C., Mastroianni N., López de Alda M., Barceló D., Valcárcel Y. (2019). Psychoactive pharmaceuticals and illicit drugs in coastal waters of North-Western Spain: Environmental exposure and risk assessment. Chemosphere.

[22] Burkina V., Zlabek V., Zamaratskaia G. (2015). Effects of pharmaceuticals present in aquatic environment on Phase I metabolism in fish. Environ. Toxicol. Pharmacol..

[23] Burkina V., Rasmussen M.K., Pilipenko N., Zamaratskaia G. (2017). Comparison of xenobiotic-metabolising human, porcine, rodent, and piscine cytochrome P450. Toxicology.

[24] Dubreil E., Sczubelek L., Burkina V., Zlabek V., Sakalli S., Zamaratskaia G., Hurtaud-Pessel D., Verdon E. (2020). In vitro investigations of the metabolism of Victoria pure blue BO dye to identify main metabolites for food control in fish. Chemosphere.

[25] Zlabek V., Burkina V., Borrisser-Pairó F., Sakalli S., Zamaratskaia G. (2016). Phase I metabolism of 3-methylindole, an environmental pollutant, by hepatic microsomes from carp (*Cyprinus carpio*) and rainbow trout (*Oncorhynchus mykiss*). Chemosphere.

[26] Richardson S.J., Bai A., Kulkarni A.A., Moghaddam M.F. (2016). Efficiency in drug discovery: Liver S9 fraction assay as a screen for metabolic stability. Drug Metab. Lett..

[27] Hazinski T.A., Noisin E., Hamon I., DeMatteo A. (1995). Sheep lung cytochrome P4501A1 (CYP1A1): cDNA cloning and transcriptional regulation by oxygen tension. J. Clin. Invest..

[28] Kim E.-Y., Iwata H., Fujise Y., Tanabe S. (2004). Searching for novel CYP members using cDNA library from a minke whale liver. Mar. Environ. Res..

[29] Loos R., Carvalho R., António D.C., Comero S., Locoro G., Tavazzi S., Paracchini B., Ghiani M., Lettieri T., Blaha L. (2013). EU-wide monitoring survey on emerging polar organic contaminants in wastewater treatment plant effluents. Water Res..

[30] Patel M., Kumar R., Kishor K., Mlsna T., Pittman C.U., Mohan D. (2019). Pharmaceuticals of emerging concern in aquatic systems: Chemistry, occurrence, effects, and removal methods. Chem. Rev..

[31] Kobayashi K., Chiba K., Yagi T., Shimada N., Taniguchi T., Horie T., Tani M., Yamamoto T., Ishizaki T., Kuroiwa Y. (1997). Identification of cytochrome P450 isoforms involved in citalopram *N*-demethylation by human liver microsomes. J. Pharm. Exp. Ther..

[32] Rochat B., Amey M., Gillet M., Meyer U.A., Baumann P. (1997). Identification of three cytochrome P450 isozymes involved in *N*-demethylation of citalopram enantiomers in human liver microsomes. Pharmacogenetics.

[33] Bae S.H., Lee J.K., Cho D.-Y., Bae S.K. (2014). Simultaneous determination of metoprolol and its metabolites, α-hydroxymetoprolol and *O*-desmethylmetoprolol, in human plasma by liquid chromatography with tandem mass spectrometry: Application to the pharmacokinetics of metoprolol associated with CYP2D6 genotypes. J. Sep. Sci..

[34] Hicks J.K., Bishop J.R., Sangkuhl K., Müller D.J., Ji Y., Leckband S.G., Leeder J.S., Graham R.L., Chiulli D.L., Llerena A. (2015). Clinical Pharmacogenetics Implementation, C., Clinical pharmacogenetics implementation consortium (CPIC) guideline for CYP2D6 and CYP2C19 genotypes and dosing of selective serotonin reuptake inhibitors. Clin. Pharmacol. Ther..

[35] Kobayashi K., Ishizuka T., Shimada N., Yoshimura Y., Kamijima K., Chiba K. (1999). Sertraline *N*-demethylation is catalyzed by multiple isoforms of human cytochrome P-450 in vitro. Drug Metabol. Dispos..

[36] Obach R.S., Cox L.M., Tremaine L.M. (2005). Sertraline is metabolized by multiple cytochrome P450 enzymes, monoamine oxidases, and glucuronyl transferases in human: An in vitro study. Drug Metabol. Dispos..

[37] Connors K.A., Du B., Fitzsimmons P.N., Hoffman A.D., Chambliss C.K., Nichols J.W., Brooks B.W. (2013). Comparative pharmaceutical metabolism by rainbow trout (*Oncorhynchus mykiss*) liver S9 fractions. Environ. Toxicol. Chem..

[38] Ereshefsky L., Dugan D. (2000). Review of the pharmacokinetics, pharmacogenetics, and drug interaction potential of antidepressants: Focus on venlafaxine. Depress. Anxiety.

[39] Burkina V., Sakalli S., Pilipenko N., Zlabek V., Zamaratskaia G. (2018). Effect of human pharmaceuticals common to aquatic environments on hepatic CYP1A and CYP3A-like activities in rainbow trout (*Oncorhynchus mykiss*): An in vitro study. Chemosphere.

[40] Huang I.J., Sirotkin H.I., McElroy A.E. (2019). Varying the exposure period and duration of neuroactive pharmaceuticals and their metabolites modulates effects on the visual motor response in zebrafish (*Danio rerio*) larvae. Neurotoxicol. Teratol..

[41] Sun Y.L., Dreier J.W., Liu X.Q., Ingstrup K.G., Mægbæk M.L., Munk-Olsen T., Christensen J. (2019). Trend of antidepressants before, during, and after pregnancy across two decades-A population-based study. Brain Behav..

[42] Howland R.H. (2012). A question about the potential cardiac toxicity of escitalopram. J. Psychosoc. Nurs. Ment. Health Serv..

[43] Piña I.L., Di Palo K.E., Ventura H.O. (2018). Psychopharmacology and cardiovascular disease. J. Am. Coll. Cardiol..

[44] Wierzbinski P. (2019). Citalopram-what you need to know about this proven antidepressant. Psychiatr. Psychol. Klin..

[45] von Moltke L.L., Greenblatt D.J., Grassi J.M., Granda B.W., Venkatakrishnan K., Duan S.X., Fogelman S.M., Harmatz J.S., Shader R.I. (1999). Citalopram and desmethylcitalopram in vitro: Human cytochromes mediating transformation, and cytochrome inhibitory effects. Biol. Psychiatry.

[46] Burkina V., Sakalli S., Zlabek V., Zamaratskaia G. (2018). CYP1A1 activity in rainbow trout is inhibited by the environmental pollutant p-cresol. Environ. Toxicol. Pharmacol..

[47] Szotakova B., Baliharova V., Lamka J., Nozinova E., Wsol V., Velik J., Machala M., Neca J., Soucek P., Susova S. (2004). Comparison of in vitro activities of biotransformation enzymes in pig, cattle, goat and sheep. Res. Vet. Sci..

[48] Maté M.L., Lifschitz A., Sallovitz J., Ballent M., Muscher A.S., Wilkens M.R., Schröder B., Lanusse C., Virkel G.L. (2012). Cytochrome P450 3A expression and function in liver and intestinal mucosa from dexamethasone-treated sheep. J. Vet. Pharmacol. Ther..

[49] Stuchlíková L., Jirásko R., Vokřál I., Lamka J., Špulák M., Holčapek M., Szotáková B., Bártíková H., Pour M., Skálová L. (2013). Investigation of the metabolism of monepantel in ovine hepatocytes by UHPLC/MS/MS. Anal. Bioanal. Chem..

[50] Smith P.K., Krohn R.I., Hermanson G.T., Mallia A.K., Gartner F.H., Provenzano M.D., Fujimoto E.K., Goeke N.M., Olson B.J., Klenk D.C. (1985). Measurement of protein using bicinchoninic acid. Anal. Biochem..

[51] Sakalli S., Burkina V., Zlabek V., Zamaratskaia G. (2015). Effects of acetone, acetonitrile, ethanol, methanol and DMSO on cytochrome P450 in rainbow trout (*Oncorhynchus mykiss*) hepatic microsomes. Toxicol. Mech. Met..

[52] Hou R., Huang C., Rao K., Xu Y., Wang Z. (2018). Characterized in vitro metabolism kinetics of alkyl organophosphate esters in fish liver and intestinal microsomes. Environ. Sci. Technol..

[53] Stiborova M., Borek-Dohalska L., Aimova D., Kotrbova V., Kukackova K., Janouchova K., Rupertova M., Ryslava H., Hudecek J., Frei E. (2006). Oxidation pattern of the anticancer drug ellipticine by hepatic microsomes-similarity between human and rat systems. Gen. Physiol. Biophys..

[54] Jonsson M.E., Brunstrom B., Ingebrigtsen K., Brandt I. (2004). Cell-specific CYP1A expression and benzo[a]pyrene adduct formation in gills of rainbow trout (*Oncorhynchus mykiss*) following CYP1A induction in the laboratory and in the field. Environ. Toxicol. Chem..

