# Supplementary material for: In Vitro Metabolic Transformation of Pharmaceuticals by Hepatic S9 Fractions from Common Carp (Cyprinus carpio)
Source: Molecules. 2020 Jun 10;25(11):2690. doi: 10.3390/molecules25112690 (PMC7321103; doi:10.3390/molecules25112690)
Supplement: Supplementary file 1 [file molecules-25-02690-s001.pdf]

Compounds	Concentration, ng/g	SD, ng/g	Frequency
10, 11 trans dihydro carbamazepine			
10, 11-dihydro carbamazepine			
Alfuzosin			
Amitryptiline			
Atenolol	0,23		1/12
Atorvastatin			
Azithromycin			
Bezafibrate			
Biperiden			
Bisoprolol			
Caffeine	2,48	3,37	12/12
Carbamazepine			
Carbamazepine 10, 11 epoxide			
Cetirizine			
Cilazapril			
Citalopram	0,15	0,04	2/12
Clarithromycin			
Clemastine			
Clindamycin			
Clindamycin sulfoxide			
Clomipramine	0,08		1/12
Clonazepam			
Diclofenac			
Dicycloverine			
Diltiazem			
Diphenhydramine			
Disopyramide			
Donepezil			
Erythromycine			
Fenbendazole	0,46	0,18	12/12
Fenofibrate			
Fexofenadine			
Glibenclamide			
Glimepiride			
Haloperidol			
Irbesartan			
Levamisole			
Loperamide	0,83	1,07	6/12
Maprotiline			
Mebendazole	7,02	1,53	12/12
Meclozine			
Memantine	0,33		1/12
Metamphetamine			
Metoprolol			
Metoprolol acid	0,24		1/12
Mianserin			
Miconazole			
Miconazole_f			
Mirtazapine			

N1-Acetylsulfamethoxazole			
N4-acetylsulfamethoxazole			
N-Desmethylcitalopram	0,17		1/12
Norsertraline			
O-Desmethylvenlafaxine			
Orphenadrine			
Oseltamivir carboxylate			
Oxazepam			
Oxcarbazepine			
Pizotifen			
Propranolol			
Ropinirole			
Roxithromycin			
Sertraline	0,12	0,09	5/12
Sotalol			
Sulfaclozine			
Sulfamethazine			
Sulfamethizole			
Sulfamethoxazole			
Telmisartan			
Terbinafine			
Terbutaline			
Toltrazuril			
Tramadol	0,26	0,03	5/12
Triamterene			
Trimethoprim			
Valsartan			
Venlafaxine	0,15		1/12
Verapamil			

LOQ min	LOQ max
0,49	0,73
0,077	0,12
0,15	0,21
0,34	0,44
0,014	0,022
0,18	0,28
1,2	1,7
0,039	0,059
0,046	0,069
0,058	0,081
1	1,7
0,13	0,2
0,46	0,69
0,027	0,041
0,039	0,06
0,11	0,16
8,4	12
0,055	0,083
0,023	0,033
0,089	0,13
0,059	0,089
0,27	0,43
4,7	7,1
0,068	0,1
0,073	0,11
0,11	0,17
0,051	0,07
0,067	0,091
0,25	0,35
0,5	0,76
0,61	0,92
0,16	0,24
0,099	0,15
0,21	0,32
0,1	0,15
0,061	0,092
0,046	0,073
0,049	0,073
0,076	0,099
5,1	7,8
0,066	0,1
0,094	0,13
0,2	0,3
0,0075	0,01
0,16	0,26
0,29	0,43
0,31	0,47
0,31	0,47
0,09	0,12

0	0
6,4	11
0,13	0,2
5,8	8,8
0,088	0,13
0,063	0,095
0,19	0,32
0,44	0,7
0,034	0,051
0,069	0,1
0,0058	0,008
0,13	0,18
0,54	0,82
0,042	0,064
0,095	0,15
0,13	0,19
0,013	0,022
0,0093	0,015
0,2	0,32
0,16	0,24
0,045	0,068
0,062	0,097
2,1	7,3
0,2	0,27
0,23	0,38
0,048	0,081
0,72	1,1
0,1	0,14
0,068	0,088
